# Prognostic Efficacy of Nuclear Morphometry at Invasive Front of Oral Squamous Cell Carcinoma: An Image Analysis Microscopic Study

**DOI:** 10.1155/2014/247853

**Published:** 2014-11-19

**Authors:** V. K. Vaishnavi Vedam, Karen Boaz, Srikant Natarajan

**Affiliations:** ^1^Department of Oral Pathology, Manipal College of Dental Sciences, Manipal University, Mangalore, Karnataka 575001, India; ^2^No. 24/1129, Padmapuram, Jyothipuram Lane, Valiachalai, Trivandrum, Kerala 695036, India

## Abstract

*Background*. Oral cancer is revisited on a pathologist perspective with advanced imaging technique. *Objective*. The present study assessed the new malignancy grading system at tumor proper (TP) and Bryne's grading system at invasive tumor front (ITF), morphometric features using software, to clarify their associations with prognosis of oral cancers. *Methods*. Histologically confirmed oral squamous cell carcinoma (OSCC) with 5-year follow-up was assessed morphometrically using image analysis at TP and ITF, correlated with the prognosis of patient. *Results*. On comparison of grading systems, a moderate agreement between both (Bryne and Anneroth) was seen. Among all histological parameters, we noted inverse correlation between degree of mitosis at invasive front and decrease in lymphoplasmacytic infiltrate at ITF with increase rate of recurrence and event of death. An increase in nuclear area, diameter, and perimeter along with decrease circularity in advancing OSCC was seen. Correlation of parameters showed higher values for maximum nuclear diameter, perimeter, and circularity at TP and ITF with recurrence. *Conclusion*. This study, while limited in sample size, concluded that a combined assessment of clinical TNM staging, histopathological grading system {excluding the parameter “mitotic activity” (due to its inverse relation)}, and nuclear morphometry at the ITF are better prognosticators. This combination proved to be an accurate predictive factor in eliciting the varied molecular characteristics of tumor heterogeneity.

## 1. Introduction

Head and neck squamous cell carcinoma (HNSCC) is an important cause of morbidity and mortality in addition to being one of the ten most common cancers occurring worldwide exhibiting marked geographic differences in occurrences [1].

Estimates based on the crude incidence rates under the National Cancer Registry Project revealed oral cancer to constitute 12% of all cancers in metropolitan cities and a frequency of 15–20% of all cancers reported from various cancer hospitals in India. Clinically, available data suggests that 60–80% of patients diagnosed with OSCC showed lymph node involvement at the time of presentation and only 10–15% of patients with OSCC presented with “localized” (T1 and T2) stages. Thus, OSCC is known to exhibit early metastasis and high degree of locoregional recurrence. This aggressive head and neck malignancy is associated with severe morbidity and lesser five-year survival rate, 32% (radiotherapy), 48% (surgery), and 54% (combined surgery and radiotherapy), despite advances in treatment [1].

OSCC constitutes a heterogeneous group of disorders exhibiting varied biological behavior and prognosis. However, till date, classical pTNM (TNM staging along with the histological grading) has been considered to be the best prognosticator of OSCC as it identifies the tumor cell morphology and its true behavior. Despite these advances, the value of histological classification of conventional OSCC remains controversial in determining the complete prognosis of an individual as few midsized tumors (approx. 3–5 cm in size) graded as well and moderately differentiated tumors by the conventional histological grading system still exhibited an aggressive behavior [2].

The cells from the deep invasive margins of the tumor are highly aggressive and less differentiated as compared to the cells at the most superficial portion of the tumor [3, 4]. Characterization of tumor cell features at the invasive front by eliciting a more objective method justifies all the features classified by the current histological grading system.

Measurement of these morphometric features (nuclear/cytoplasmic ratio, nuclear area, nuclear perimeter, and circularity) can be done either manually using ocular grids or objectively with using computer-based image analysis (greater accuracy) that traces microscopic images and measures them in an objective and reproducible manner [5].

This study investigated the tumor features using the Bryne grading system at ITF and Anneroth grading system at TP in various histological grades of OSCC (well differentiated, moderately differentiated, and poorly differentiated) and correlated the same with the prognosis of the patient. An attempt has also been made to assess morphometric features using the Image J software and results were compared with clinical and histopathological findings to clarify their associations with treatment and prognosis.

## 2. Material and Methods

Thirty-two archival biopsy specimens of histologically proven OSCC were diagnosed and critically analyzed for a five-year survival rate at the Department of Oral Pathology and Microbiology, Manipal College of Dental Sciences, Mangalore, which were considered after an institutional ethical committee clearance was obtained. The subject group selected was cases of primary OSCC with the least available follow-up of five years (alive with/without OSCC, dead due to OSCC) from the initial diagnosis. The study excluded patients who died due to reasons other than OSCC, those who underwent any form of treatment other than surgery, and OSCC patients who did not complete the entire course of the treatment. The control group comprised tissues from buccal mucosa obtained post mortem from ten individuals with no clinically apparent oral lesions.

The clinical data regarding age, gender, site, and clinical presentation of tumor and TNM stage were recorded from the patient's files. The treatment modality rendered to the patient (surgery) was considered along with histological diagnosis to determine the prognosis of the patient (*comparison of surgery performance date to the present state of survival at the time of study conducted by telephonic conversation and letters*).

### 2.1. Procedure

For each case, two serial sections of four-micrometer thickness were taken from formalin-fixed, paraffin-embedded representative sites from the tissues of OSCC obtained from the Department of Oral Pathology and Microbiology, Manipal College of Dental Sciences, Mangalore. Similarly two serial sections were taken from formalin-fixed, paraffin-embedded tissues of normal buccal mucosa to serve as controls. One section each was stained with hematoxylin and eosin stain (to histologically grade the ITF as per the grading system proposed by Bryne et al.; superficial/other areas of the tumor were evaluated using grading system of Anneroth et al.). The next serial tissue section was stained using the Feulgen stain to assess the nuclear morphometry at ITF using* Image J 1.42q image analysis* software. We assessed the individual histological grades at tumor proper and invasive front OSCC cases using the grading system (Table 1).

#### 2.1.1. Histological Grading of Malignancy of Tumor Cell Population

The* degree of keratinisation*,* nuclear pleomorphism*, number* of mitoses*,* pattern of invasion*, and* lymphoplasmacytic infiltration* were assessed in a maximum of ten nonoverlapping, contiguous microscopic fields at tumor proper and invasive front per case.

This procedure of the histological grading was done at invasive front of the tumor (Bryne) and superficial/other parts of the tumor (Anneroth) with the similar parameters as mentioned in Table 1. Data was entered into the standard Excel format created (ten fields for each of the parameters) and average was evaluated for each parameter. The sum of all these parameters at tumor proper and invasive front was calculated to obtain the final scores and graded accordingly (grading systems Anneroth and Bryne, resp.) (Table 2).

### 2.2. Procedure for Image Analysis (Pictorial Diagram: see (Figure 6))

**Figure 1:**
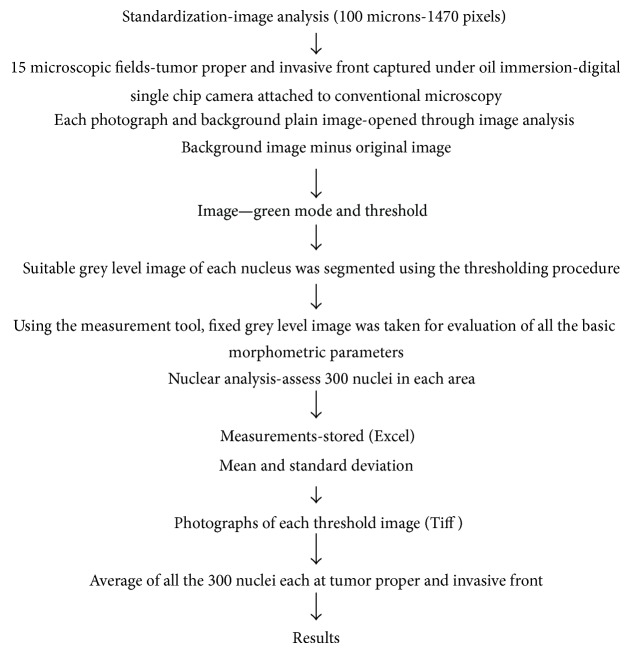
Stepwise description of Image J analysis.

Image J software analysis was used to assess the cellular and nuclear measurements on Feulgen-stained sections quantitatively. The Feulgen-stained sections appeared magenta pink or reddish purple under oil immersion objective (Leitz HM Lux-3 light microscope). Procedure is done in a stepwise manner (Figure 1).

Nuclei of epithelial tumor cells with well demarcated, clean, and nonoverlapping borders with absence of pyknosis were selected. The selected nuclei were assessed for the following features: nuclear diameter (in microns), nuclear area (in square microns), nuclear perimeter (in microns), and shape description (circularity graded on scale of 0 {a line}-1 {a circle}) [9].

Using all the above histological parameters as graded by Anneroth et al. at tumor proper/Bryne et al. at invasive front and nuclear morphometric parameters, image analysis, Chi-square test, Student's *t*-test, and Mann-Whitney analysis were performed to assess the individual parameters and combination of histological and morphometric parameters with the prognosis of the patient.

## 3. Results

### 3.1. Comparison of Morphometric Parameters between Cases and Control

Nuclear morphometric parameters (nuclear area, minimum diameter, maximum diameter, perimeter, and circularity) were compared at tumor proper and ITF between 32 cases and 10 controls. Using Mann-Whitney analysis, we observed that circularity of cells at tumor proper (*Z* = 2.303; *P* = 0.021) was statistically different between cases and controls where cells were more circular of value towards 1 (mean values 0-1) and uniform in controls. However, we also found that parameters like nuclear area, minimum diameter, perimeter, and circularity were greater in cell nuclei of OSCC patients as compared to controls of normal buccal mucosa. Among all the parameters, nuclei circularity was observed to become less in carcinoma patients thus indicating more elliptical nature of nuclei in OSCC cells.

### 3.2. Histological Grades Obtained by Grading System Proposed by Anneroth and Bryne

Based on our grading of various parameters, 32 cases were divided into well differentiated, moderately differentiated, and poorly differentiated carcinoma with a frequency of 15, 13, and 04 cases, respectively, based on grading system given by Anneroth et al. Likewise, 10, 14, and 08 cases of OSCC were graded as well differentiated, moderately differentiated, and poorly differentiated carcinoma according to grading system of Bryne et al. We observed that, among the 32 cases of OSCC evaluated, the majority of cases (35.7%) were well differentiated by Anneroth et al. whereas, as per Bryne grading system, the majority of these same cases (33.3%) were categorized as moderately differentiated.

### 3.3. Comparison of Histological Grading with Recurrence Event

According to Anneroth grading system we observed that 81.81% (well differentiated), 77.77% (moderately differentiated), and** 50%** (poorly differentiated) cases exhibited recurrences. According to Bryne grading system we observed that 80% (well differentiated), 72.72% (moderately differentiated), and** 75%** (poorly differentiated) cases exhibited recurrences. On comparison between both grading systems, it was evident that a greater percentage,** 75%,** of cases were graded as poorly differentiated carcinoma by Bryne's grading system, as compared with** 50%** of cases in Anneroth grading system which showed recurrence, thus proving Anneroth's grading system under diagnosis of the true grade of the tumor.

### 3.4. Comparison of Grading System Proposed by Anneroth with Survival Event

According to Anneroth et al. grading system we observed that 28.57% (well differentiated), 33.33% (moderately differentiated), and 33.33% (poorly differentiated) cases exhibited increased rate of survival in future. According to Bryne grading system we observed that 33.33% (well differentiated), 14.28% (moderately differentiated), and 50% (poorly differentiated) cases exhibited survival. On comparison between both grading systems, it was evident that a greater percentage,** 50%,** of cases were graded as poorly differentiated carcinoma as compared with** 33.33%** of cases in Anneroth grading system correlated with degree of survival event, thus proving its Bryne grading accuracy in identification of survival rate.

### 3.5. Correlation between Anneroth's and Bryne's Grading Systems

We used kappa statistics to correlate the extent of agreement between Anneroth and Bryne grading systems. Among all the cases assessed both grading systems agreed upon 6 cases of well differentiated, 8 cases of moderately differentiated, and 3 cases of poorly differentiated squamous cell carcinoma. This showed that Anneroth and Bryne et al. agreed over 51% (fair) cases with statistically significant value (kappa value: 0.273; *P* = 0.035).

### 3.6. Assessment of Morphometric Parameters with Grading System Proposed by Anneroth

**Figure 2:**
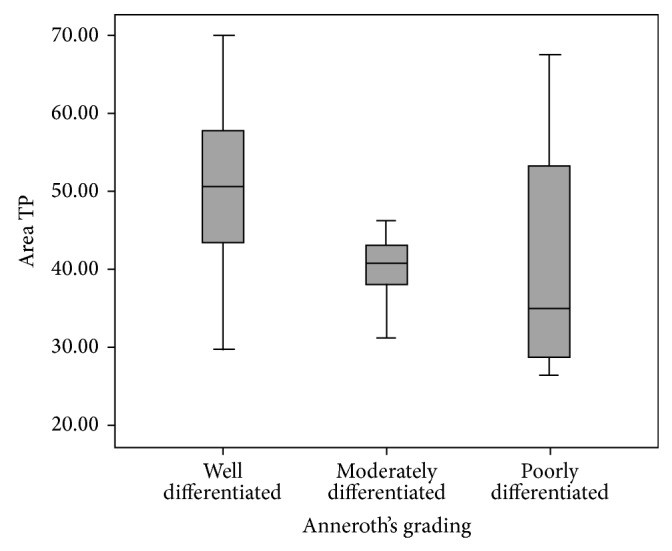
This graph depicts the relation of nuclear area at tumor proper with Anneroth et al. grading system in oral squamous cell carcinoma patients (graph depicted “tumour proper as TP”) (*n* = 32). The statistical significance was calculated using Chi-square test (value = 6.686) and statistical significance shown as ^∗^ *P* = 0.035.

**Figure 3:**
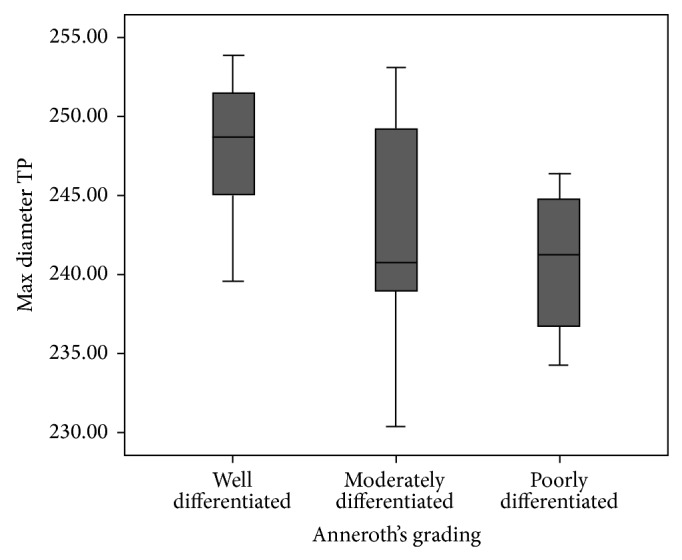
This graph depicts the relation of maximum diameter at tumor proper with Anneroth et al. grading in oral squamous cell carcinoma patients (graph depicted “maximum diameter tumor proper as Max diameter TP”) (*n* = 32). The statistical significance was calculated using Chi-square test (value = 7.351) and statistical significance shown as ^∗^ *P* = 0.025.

On statistical analysis, we observed that nuclear area and maximum diameter at tumor proper were significantly different between well differentiated, moderately differentiated, and poorly differentiated squamous cell carcinoma patients with Chi-square value 6.686 and *P* = 0.035 (Figure 2) and Chi-square value 7.351 and *P* = 0.025 value (Figure 3), respectively. However, among all the parameters, as the grades of malignancy increased, a decrease in nuclear area and perimeter and an increase in circularity were noticed.

### 3.7. Assessment of Morphometric Parameters with Bryne Grading

**Figure 4:**
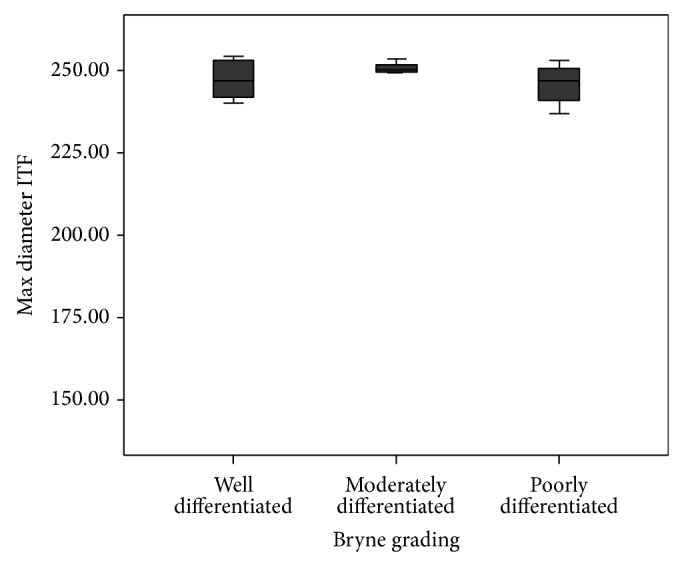
This graph depicts the relation of maximum diameter at invasive front with Bryne et al. grading in oral squamous cell carcinoma patients (graph depicted “maximum diameter invasive tumour front as Max diameter ITF”) (*n* = 32). The statistical significance was calculated using Chi-square test and compared.

**Figure 5:**
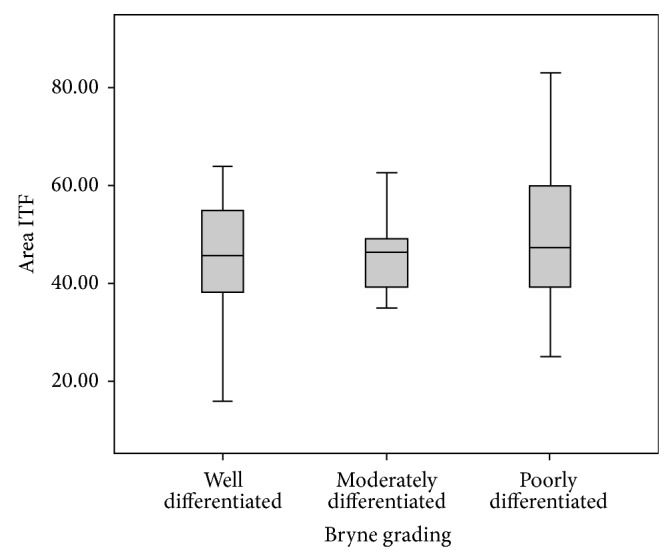
This graph depicts the relation of nuclear area at invasive front with Bryne et al. grading in oral squamous cell carcinoma patients (graph depicted “area tumour proper as Area TP”) (*n* = 32). The statistical significance was calculated using Chi-square test and compared.

We observed that maximum diameter (Figure 4) and nuclear area (Figure 5) at invasive front were higher in poorly differentiated squamous cell carcinoma. On the contrary, circularity decreased with increase in histological grades of malignancy. This elicits that there was a significant increase in the values from well differentiated to poorly differentiated squamous cell carcinoma in nuclear area in Bryne grading system in comparison to Anneroth et al. grading system.

### 3.8. Assessment of Morphometry with Recurrence

Few parameters like nuclear maximum diameter, perimeter, and circularity, both at the tumor proper and at invasive front area, were found to be higher in patients with observed recurrences with OSCC.

### 3.9. Assessment of Morphometry with Survival

An increase in maximum diameter and perimeter and a decrease in minimum diameter with increase in different grades of malignancy at tumor proper of OSCC patients were observed. However, increases in values of nuclear area and maximum diameter were observed at invasive front in OSCC patients.

### 3.10. Comparison of Individual Histological Grades

On evaluation of individual histological grades both at tumor proper and at invasive front with the recurrence, we noticed that, among other parameters, there was a significant decrease in number of mitosis at tumor proper (*z* value = −2.022; *P* value = 0.047). The degree of keratinization and host response decreased in cases of recurrence and increase in the rate of pleomorphism with progressive increase in the recurrence event in patients exhibiting OSCC. This also elicited an increase in the total score of Bryne grading system as compared to Anneroth grading system in OSCC patients with recurrences.

On comparison of individual histological parameters with the degree of survival event, degree of keratinization at the invasive front of the tumor was only found to be statistically significant (*z* value = −2.027; *P* = 0.027).

### 3.11. Assessment of T, N, M, and TNM Staging with Recurrence

We observed the individual tumor, node, and TNM staging with recurrences using Chi-square test. Among these parameters, individual tumor factor and combined TNM staging exhibited statistically significant results of *F* value = 7.180 and *P* = 0.040 and *F* value = 8.363 and *P* = 0.024, respectively. We also noticed that a progressive increase from T1 to T3 tumor status and lymph node involvement (number and size) correlated directly with the recurrence event. Also, stage 3 (100.0%) and stage 4 (57.1%) patients presented with increased susceptibility to carcinoma recurrence as compared to stage 1 and 2 carcinoma patients.* Note*. Metastasis value was not assessed due to inadequate clinical data available to incorporate into the system for statistical analysis.

### 3.12. Assessment of T, N, M, and TNM Staging with Survival

The individual clinical grades of tumor, nodal status, and combined TNM staging with the survival event in oral squamous cell carcinoma patients were analyzed. However, none of the above parameters correlated well with survival of the patients. From the above analysis, it was evident that there was a progressive increase in a small proportion of the patients with stage 3 and 4 TNM staging correlated directly with high degree of mortality.* Note*. Metastasis value was not assessed due to inadequate clinical data available to incorporate into the system for statistical analysis.

### 3.13. Combination of Morphometry and Individual Parameters with Recurrence

Forward stepwise linear regression with morphometric parameters and the degree of mitosis at tumor proper could be used as a predictor of recurrence with statistical significant value of *T* value = −2.105; *P* = 0.047 RECURRENCE = 0.872 – 0.158 (MITOSIS).

### 3.14. Assessment of Individual Histological Grades, Morphometry, and Survival

On performing forward stepwise linear regression analysis, we observed that circularity at ITF (*F* value = 9.422; *P* = 0.012) and a combination of both circularity and maximum diameter at invasive front (*F* value = 10.223; *P* = 0.005) could be used to assess the survival of the patients.

### 3.15. Assessment of Individual Histological Grades, Morphometry, and Recurrence

From the above analysis, we observed that combined degree of mitosis at tumor proper and host response at invasive front (*F* value = 7.333; *P* = 0.005) were the best predictors of recurrences in OSCC patients.

## 4. Discussion

Presently, the New Malignancy Grading System (as proposed by Anneroth et al.) [6, 10] and its modification with the elimination of parameter titled “mode of invasion” (as suggested by Bryne et al.) [8, 11, 12] are considered to be better prognostic indicators in OSCC as used at the tumor proper and ITF, respectively. However, few cases graded as well differentiated tumors have turned lethal later in the progress of the disease. Morphometry (*computerized image analysis*) is proven to be an objective prognosticator in cancers of breast. With this background, the present study assessed and correlated the established grading systems and nuclear morphometry using image analysis at the invasive front of tumor and at tumor proper and correlated the same with the prognosis of OSCC patients.

Comparison of the specificity of grading systems (*suggested by* Bryne et al.* and* Anneroth et al.) in the study showed the presence of moderate agreement between the grading systems in about 51% of the cases (17 out of 32 cases; kappa value = 0.273; *P* = 0.035) that failed to demonstrate unequivocally the better system for grading OSCC. This is in agreement with experiments by Weijers who found that the New Malignancy Grading System was no better than the conventional grading system (Anneroth) in predicting the prognosis of small squamous cell carcinoma of tongue and floor of the mouth. However, in contradiction, Bryne et al. have reported higher reproducibility and greater levels of agreement with invasive front grading in comparison with previous grading systems [7].

On assessment of recurrence and survival using the grading systems by Bryne and Anneroth, we observed that 75% of cases of OSCC graded as poorly differentiated by grading system of Bryne exhibited recurrence in comparison to 50% of cases similarly graded as per criteria of Anneroth et al. Survival analysis showed that 50% of cases deemed poorly differentiated by system of Bryne et al. died due to OSCC in contrast to 33.33% of cases graded as poorly differentiated by the grading system of Anneroth. Thus, this demonstrates, in terms of better prognostication, that invasive front grading (IFG) system by Bryne does not inaccurately diagnose the tumor to advanced grades. This could be attributed to the differential behaviour of tumor cells at the invasive front of OSCC [4].

On evaluation of individual histological parameters, we noted that degree of keratinization decreased with recurrence and event of death. Similar results were observed by Anneroth and Hansen who showed a negative correlation between “*tendency to keratinization*” and the total malignancy score in carcinomas of tongue and floor of the mouth [13].

Using linear regression analysis, we observed a marked decrease in the amount of lymphoplasmacytic infiltrate at tumor proper and ITF with increased recurrence and event of death of patients with OSCC. This was in accordance with studies by Anneroth and Hansen, who stated that the presence of inflammation may be due to the expression of an immunologic reaction against the tumor and thus appeared to be a sign of relative benignancy. The presence of high degree of tumor-associated tissue eosinophilia (TATE) provides good host-tissue protective response, thereby increasing the survival rate of the patient [14].

Among other histological parameters, we noted an inverse correlation between degree of mitosis at invasive front and rate of recurrence and event of death. Bryne et al. stated that low mitotic count may be seen at the invasive front of the tumor as compared to solid areas of the tumor with high dissociation of cells in the most invasive parts of tumor [15, 16]. In addition, Bryne et al. (in the same study) also assessed the prognosis of OSCC patients by separately grading the ITF with “inclusion” and subsequently by “omission” of the parameter of “mitotic count.” The prognostic value still remained highly significant and reproducibility improved when the parameter of “mitotic count” was eliminated from IFG [16].

However, several studies have demonstrated an increased frequency of mitoses with increase in grades of malignancy [17, 18]. Further, analysis by Crissman et al. stated that increased frequency of mitoses correlated directly with poor survival [19]. This variation (*P* = 0.047) of the parameter of “mitotic activity” among studies could be attributed to the tumor heterogeneity, interobserver disagreement, and variations in the size of the high power fields in different microscopes rendering the studies not comparable.

In the present study, computerized image analysis of nuclear morphometric features like area, minimum diameter, and perimeter showed an* increase* in histological sections of OSCC as compared to normal buccal mucosa (control) in concordance with the study by van der Wal et al. and Kinoshita et al. [9, 15].

We also noticed a gradual* decrease* in nuclear area with increase in grades of malignancy. This may be attributed to the fact as stated from earlier studies by Pereira et al. that initially nuclear and cellular volume always increases first, followed by a reduction in nuclear and cell ratio as the cell progresses from dysplastic stage towards carcinoma [14]. However, nuclear/cytoplasmic size increases to higher value in malignant cells in comparison to normal cells. Also, Franklin and Smith illustrated that tumor cell nuclei with similar nuclear DNA content exhibited a wide and larger range of nuclear size as compared with normal cells [20].

An inverse relation between increased nuclear perimeter and loss of differentiation was noted in our study where we found a progressive increase in nuclear perimeter at invasive front from well differentiated to poorly differentiated squamous cell carcinoma. This is in agreement with the findings of Kinoshita et al. who in a combined study of 130 cases of precancerous and cancerous lesions found a progressive increase in nuclear DNA content, nuclear area, and perimeter at tumor proper that related directly to the proliferation of cells [15, 20].

Among the other nuclear parameters assessed in the present study, decrease in numerical values pertaining to nuclear circularity was observed indicating a transition from normal oral mucosa to malignancy. These findings reflect those reported by Abdel-Salam et al. who in a study of 16 oral epithelial lesions observed a deviation of circularity in malignancies as compared to nontransformed lesions [21]. Nuclear changes and anaplasia make the tumor cells [9] appear more spindled in carcinoma which is rightly assessed by nuclear circularity factor.

Correlation of nuclear morphometric parameters (*between TP and ITF*) with prognosis showed higher numerical values for maximum diameter of nucleus, nuclear perimeter, and circularity, both at the tumor proper and at invasive front in patients with recurrence of OSCC.

The present study therefore surmised that a combined assessment of the histological grading system and nuclear morphometric analysis at invasive front may prove to be an ideal prognosticator of OSCC.

## 5. Conclusion 

The present study aimed to assess the histopathological parameters (*based on the histological grading systems as proposed by* Anneroth et al.* used at TP and that of* Bryne et al.* at ITF*) and correlated the same with the prognosis of 32 patients diagnosed with OSCC and treated with a minimum follow-up of 5 years. Computerized image analysis software (Image J) was used to quantify the nuclear morphological parameters (nuclear area, perimeter, diameter, and circularity) and correlate the same with the survival of patients.

This study proved that histological grading system as proposed by Bryne et al. did not differ much from the grading system of Anneroth et al. in assessing the tumor characteristics. However, an “over-diagnosis” made by Bryne et al. in grading the tumor to advanced grades correlated better with the local recurrence and survival of patients. To date, prediction of prognosis using histological grading systems alone remains questionable in terms of accuracy and credibility. Hence, nuclear morphometric measurements made using image analysis software are deemed to be an objective and reproducible tool in anticipating the survival of patients. Using the regression analysis, we obtained [RECURRENCE = 1.698 − 0.236 (MIT-ANNE) − 0.335 (HOSTRES-BRY)] and [SURVIVAL EVENT = −14.021 + (14.479 (CIR ITF)) + 0.044 (MAX DIAITF)] of the nucleus to be the formula for prediction of prognosis, thus emphasizing the importance of host response, nuclear circularity, and maximum diameter of cells to be the ideal prognosticators in OSCC.

The present study, while limited in its sample size, concluded that a combined assessment of clinical TNM staging, histopathological grading system {excluding the parameter titled “mitotic activity” (due to its inverse relation)}, and nuclear morphometry at the invasive front of the tumor are better prognosticators than individual parameters. This combination proved to be a more accurate predictive factor as it is capable of eliciting varied molecular characteristics of tumor heterogeneity.

## Figures and Tables

**Figure 6:**
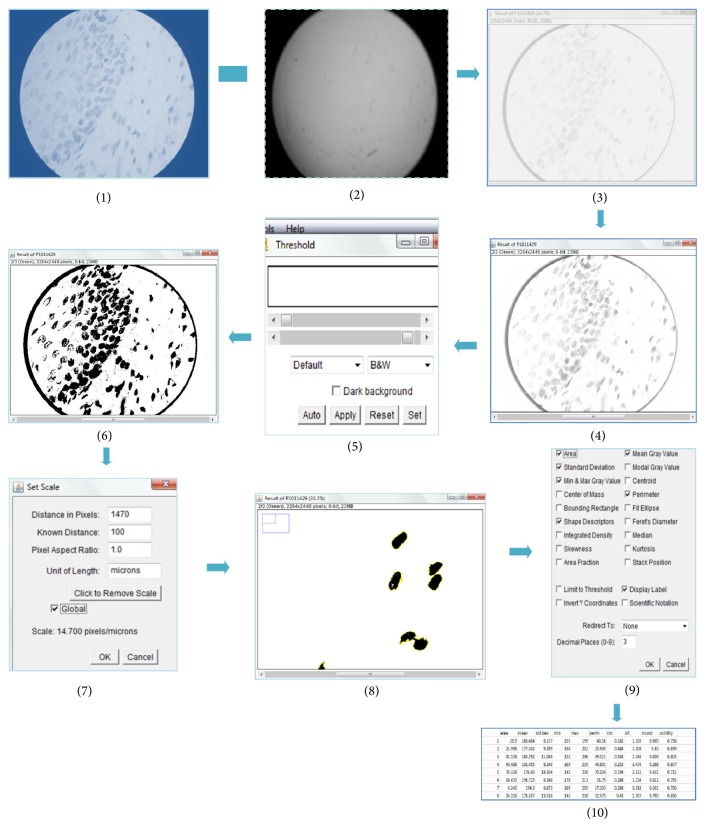
This pictorial diagram represents the stepwise procedure of image analysis after standardization. Subtract picture (1) from (2) to obtain a subtracted image (3). Subtracted image is converted to a grey level image (4) in step 2. Set the threshold (Figure (5)). Image (4) is thresholded (picture (6)). Then set the scale (picture (7)). Mark the nuclei using Wand tool (picture (8)). Set the measurements as depicted (picture (9)). Save measurements in an Excel format (picture (10)).

**Table 1:** This table depicts the new malignancy grading system of oral squamous cell carcinoma patients as proposed by Anneroth et al. (1986) [6] and Bryne et al. (1989 and 1991) [7, 8].

Morphologic parameter	Histological grading of malignancy of tumor cell population			
	1	2	3	4
Degree of keratinization	Highly keratinized (>50% of the cells)	Moderately keratinized (20–50% of the cells)	Minimal keratinization (5–20% of the cells)	No keratinization (0–5% of the cells)

Nuclear polymorphism	Little nuclear polymorphism (>75% mature cells)	Moderately abundant nuclear polymorphism (50–75% mature cells)	Abundant nuclear polymorphism (25–50% mature cells)	Extreme nuclear polymorphism (0–25% mature cells)

Number of mitoses/HPF	0-1	2-3	4-5	>5

Pattern of invasion	Pushing well-delineated infiltrating borders	Infiltrating, solid cords, bands and/or strands	Small groups or cords of infiltrating cells (*n* > 15)	Marked and wide spread cellular dissociation in small groups of cells (*n* < 15) and/or in single cells

Lymphoplasmacytic infiltration	Marked	Moderate	Slight	None

**Table 2:** Bryne et al. grading system (Bryne et al., 1989) [7].

Score	Grade
5–8	Good grade
9–12	Moderate grade
13–16	Poor grade

## Abbreviations

MIT-ANNE: Anneroth mitosis
HOSTRES-BRY: Bryne host response
CIR-ITF: Circularity invasive tumor front
MAX DIAITF: Maximum diameter Invasive tumor front.
